# Wet beriberi with multiple organ failure remarkably reversed by thiamine administration: A case report and literature review

**DOI:** 10.1097/MD.0000000000010010

**Published:** 2018-03-02

**Authors:** Yuanli Lei, Ming-Hua Zheng, Weijian Huang, Jie Zhang, Yingru Lu

**Affiliations:** a Department of Emergency Medicine; b Department of Hepatology, NAFLD Research Centre; c Department of Cardiology; d Department of Critical Care Medicine, The First Affiliated Hospital of Wenzhou Medical University, Wenzhou, China.

**Keywords:** alcohol, multiple organ failure, prison, thiamine, wet beriberi

## Abstract

**Rationale:**

Circulatory failure, especially with low systemic vascular resistance (SVR), as observed in septic shock, thyrotoxicosis, and anemia, is a particular pattern that should suggest thiamine (vitamin B1) deficiency. The clinical picture of wet beriberi secondary to thiamine deficiency only demonstrates non-specific clinical manifestations. For a diagnosis of wet beriberi, medical history is very important. Interestingly, imprisonment was also found to be related to thiamine deficiency. This article presents a rare case of wet beriberi associated with multiple organ failure (MOF) in a prison patient with years of heavy alcohol consumption.

**Patient concerns:**

The patient reported repetitive symptoms of nausea, vomiting, respiratory distress, and palpitations for a period of 1 month; dyspnea and edema for 5 days; and decreased blood pressure and urine volume for 2 days.

**Diagnoses:**

The heart failure patient had a history of dietary deficiency. Right heart catheterization showed high cardiac output (CO) and low SVR. Measurement of serum thiamine concentration was low. The most important factor was that the hemodynamic indices were remarkably reversed by thiamine administration.

**Interventions:**

The patient started treatment with thiamine (100 mg) by intramuscular injection, together with basic supportive care.

**Outcomes:**

The hemodynamic indices improved within 12 hours after thiamine administration. Echocardiographic examinations revealed right ventricular function improvement within a few days, which were normal within a month.

**Lessons:**

A diagnosis of wet beriberi should be considered for a prison patient who has unexplained heart failure, lactic acidosis, and/or MOF. Moreover, the patient should be empirically given thiamine administration without delay.

## Abstract 2

Supplemental Digital Content is available in the text

## 1 Introduction

Wet beriberi, secondary to thiamine (vitamin B1) deficiency, is characterized by cardiovascular damage, and it is different from dry beriberi, which predominantly shows neurological involvement. We found that the vast majority of wet beriberi patients have causal factors, which is key for diagnosis. Here, we reported a case of a 39-year-old man with wet beriberi who had a history of long-term drinking, imprisonment, and furosemide administration and presented with multiple organ failure (MOF). The hemodynamic indices of the patient were remarkably reversed by thiamine administration. Importantly, we reviewed the literature on wet beriberi and summarized the etiology, reports of cardiac output (CO) and systemic vascular resistance (SVR), currently available diagnostic tests, and thiamine use in patients with wet beriberi. We believe the relevant content may have important clinical implications.

## 2 Case presentation

A 39-year-old man was admitted to our hospital complaining of repetitive symptoms of nausea, vomiting, respiratory distress, and palpitations for a period of 1 month; dyspnea and edema for 5 days; and decreased blood pressure and urine volume for 2 days. Ultimately, he was referred to a local hospital with the tentative diagnosis of heart failure and was initially treated by furosemide. The patient was imprisoned for violence for 4 months, during which his body weight decreased by 15 kg. His medical history included a bilateral congenital foot deformity and heavy alcohol abuse (2500–3000 mL beer/d for 19 years). Following admission to our emergency room, a physical examination revealed the following: consciousness; temperature: 36.6 °C; blood pressure: 85/47 mmHg; heart rate: 126 bpm; body mass index: 20.5 kg/cm^2^; no mottling; no sensory or motor deficits; no nystagmus, ataxia, or paresis; dilated jugular veins with generalized pitting edema; 2 lungs that sounded clear but had left-sided hypoinflation; no obvious rales; normal heart sounds; and normal physiological reflection without pathological reflection. The laboratory data are shown in Table 1.

**Table 1:**
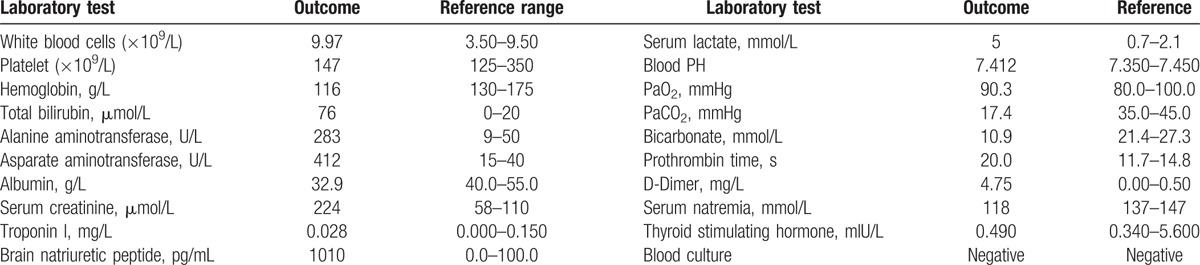
The laboratory data upon admission.

An electrocardiogram indicated sinus tachycardia. Chest computed tomography showed bilateral pleural effusion with left lung atelectasis. Transthoracic echocardiography revealed a left ventricular ejection fraction (LVEF) of 77.1% with dilatation of the left atrium cavity (the inner diameter was 44 cm and the volume was 87 mL), normal right atrial and ventricular size, tricuspid regurgitation in association with a moderate degree of pulmonary hypertension (estimated systolic pulmonary artery pressure of 57 mmHg), velocity Doppler mitral valve (i.e., *E*) of 1.55 m/s, velocity tissular Doppler mitral annulus (i.e., *E*′) of 0.09 m/s, output cardiac index by subaortic velocity time integral of 5.59 L/min/m^2^, and a small amount of pericardial effusion. The computed tomography pulmonary angiographic results were negative. Right heart catheterization showed high CO and low SVR (Table 2). Thiamine deficiency was confirmed by the blood concentration of thiamine, which was assayed as 11 ng/mL (normal range, 20–60 ng/mL).

**Table 2:**
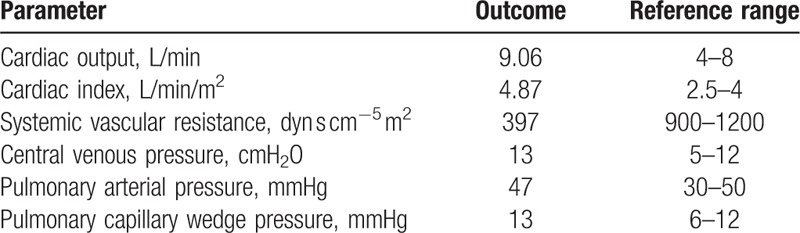
The right heart catheterization data upon admission.

**Figure 1:**
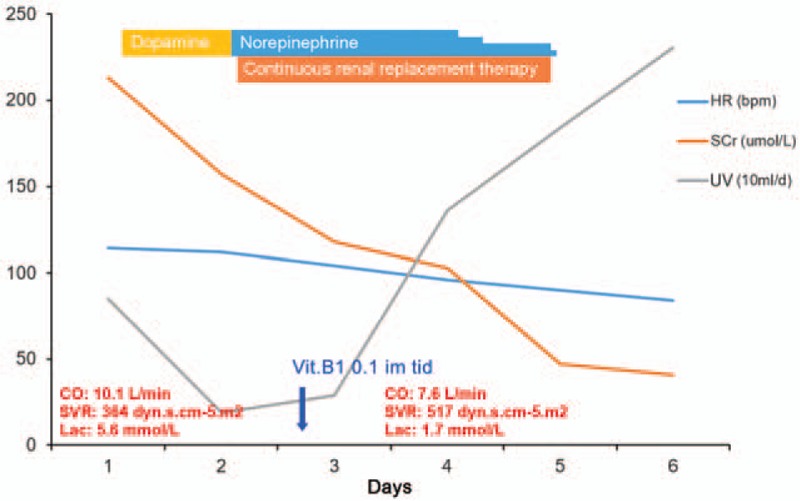
The change before and after treatment with thiamine. Vit.B1 0.1 im tid: treatment with 100 mg of vitamin B1 via intramuscular injection 3 times a day. CO = cardiac output, HR = heart rate, Lac = lactate, Scr = serum creatinine, SVR = systemic vascular resistance, UV = urine volume.

The day after admission to the emergency intensive care unit, the patient started treatment with approximately 2 L fluid challenge every day. Additionally, dopamine and norepinephrine were used to increase blood pressure. Due to high creatinine, low urine volume and generalized severe edema, continuous renal replacement therapy (CRRT) was used, together with basic supportive care. The patient was admitted to local hospital because of heart failure; the temperature of the patient was normal; the white blood cell was not high; and we cannot find the focus of the infection in the available evidence. Therefore, antibiotic administration was not initiated. On the third day, a course of vitamin B1 (100 mg) by intramuscular injection 3 times a day and 30 mg of oral vitamin B1 3 times a day was initiated. The hemodynamic indices improved within 12 hours after the first dose of thiamine injection, and we stopped vasopressor treatment and CRRT during the following 48 hours (Fig. 1, Supplementary Table 1). Heart failure symptoms (dyspnea, dizziness, etc.) disappeared as the hemodynamic status improved. On the sixth day, the injection dose was decreased to 100 mg of intramuscular vitamin B1 each day. A month later, echocardiographic examinations were normal. The patient was discharged uneventfully after 1 month of admission.

## 3 Discussion

Wet beriberi is one of the clinical syndromes associated with thiamine deficiency. Thiamine, in its phosphorylated form thiamine pyrophosphate (TPP), is the precursor for the cofactor of both pyruvate dehydrogenase and alpha-ketoglutarate dehydrogenase, which are both key enzymes of the Krebs cycle. The Krebs cycle is an essential part of aerobic glucose metabolism. A decrease in the activity of these 2 enzymes due to thiamine deficiency may lead to the tissue accumulation of pyruvate and lactate.^[1]^ Moreover, the accumulation of pyruvate and lactate decreases peripheral resistance and increases venous blood flow, increasing the cardiac preload. Increased preload and myocardial dysfunction ultimately leads to congestive heart failure.^[2,3]^ Wet beriberi mainly triggers right heart failure.^[4–6]^ The moderate pulmonary hypertension is common for wet beriberi patient.^[3,4,7]^ Our patient had a pulmonary hypertension of 57 mmHg. Increased pulmonary arterial blood flow, elevated pulmonary capillary wedge pressure reflecting elevated left ventricular end-diastolic pressure, and elevated pulmonary vascular resistance can all cause pulmonary hypertension.^[3,4]^ Hyperkinetic LVEF is observed in many patients. Attas et al^[2]^ reported that the LVEF of a wet beriberi patient was 72%; Yamamura et al^[3]^ reported the LVEF was 85%; our patient had a LVEF of 77.1%. Certain patients, including ours, may not demonstrate acidosis even in the setting of elevated lactate. The PH of these patients was preserved by a compensatory hyperventilation response.^[3,8–10]^

The store of thiamine in the body is small (approximately 30 mg), and with a half-life of 10 to 18 days.^[11–14]^ Thiamine deficiency should be suspected in patients with unexplained heart failure and lactic acidosis, in the setting of alcoholism, chronic malnutrition, and so on.^[1]^Based on the standard for wet beriberi,^[2]^ wet beriberi was established in our patient. Besides this, wet beriberi has a rare and severe form; Shoshin beriberi is an acute and fulminant form of wet beriberi, which is described as a “rapidly curable hemodynamic disaster” that is characterized by hypotension, tachycardia, and lactic acidosis.^[1,5,7,8,11,12]^ The patient's condition improved after thiamine administration, vasopressor treatment, CRRT, and so on, but without antibiotic administration. Measurement of serum thiamine concentration was low. These further confirmed that the patient was diagnosed with wet beriberi.

**Figure 2:**
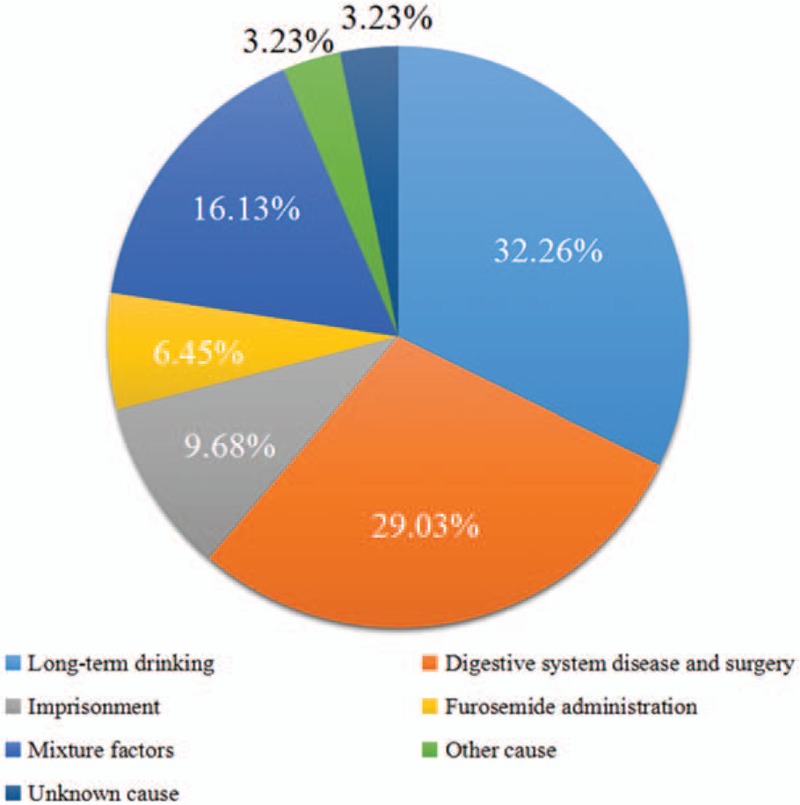
Etiology of beriberi. From 24 documents including 31 cases. Long-term drinking, digestive system disease and surgery, imprisonment, and furosemide administration were reported as causal factors. Mixture factors mean >1 factor, and at least include one of the factors mentioned above. Other cause was type-1 glycogen storage disease, which was reported in 1 case, and there was 1 case with unknown cause because the author did not mention it in the document.

For a diagnosis of wet beriberi, the patient's medical history is very important. The etiology of wet beriberi is illustrated in Fig. 2.^[1–5,7–25]^ Long-term drinking can lead to decreased vitamin B1 absorption and storage dysfunction and can increase the amount of damage; long-term drinking was the most common cause of wet beriberi. According to whether the patient has a history of long-term drinking or not, beriberi can be divided into alcohol-related beriberi and non-alcohol-related beriberi. Digestive system diseases and surgery were the other major causes of thiamine deficiency. Notably, bariatric surgery (including gastric bypass, sleeve gastrectomy, and duodenal switch) is the most important cause of vitamin B deficiency (mainly thiamine deficiency) to date.^[26]^ Interestingly, imprisonment was also found to be related to thiamine deficiency. Zhang et al^[15]^ reported 3 cases of wet beriberi in prison and mentioned that all of the prisoners had various symptoms of thiamine deficiency. Park et al^[4]^ also reported on wet beriberi in prisons in western society. Cisse et al^[27]^ studied Guinean prisons from 2010 to 2014 and found 38 cases of beriberi secondary to thiamine deficiency. There were 14 cases of demonstrated wet beriberi and 2 cases of Shoshin beriberi. Heart failure was a common observation in patients with wet beriberi. Furosemide is a loop diuretic that increases urinary output, thereby reducing edema in patients with congestive heart failure. Furosemide administration is related to thiamine deprivation, as it causes increased urinary thiamine excretion and is thus frequently associated with low thiamine intake levels.^[8,16,28]^ Furthermore, in the largest clinical study of congestive heart failure patients, 30% of patients presented with thiamine deficiency that resulted from furosemide administration.^[29]^

Even though thiamine deficiency is a non-uncommon situation in critically ill patients, wet beriberi is easily misdiagnosed and missed diagnosis. First, the typical features of wet beriberi are high CO and low SVR, but the typical features are often absent in many patients (Table 3^[2,3,5,7–9,17–19]^). Second, the clinical picture demonstrates only non-specific clinical manifestations. Some patients come to the hospital with low CO, as mentioned above, some present with ST segment elevation acute myocardial infarction,^[11,18]^ non-ST segment elevation acute myocardial infarction,^[7]^ pericardial effusion,^[3]^ or severe pulmonary arterial hypertension.^[4]^ MOF is not uncommon.^[19,20]^ Acute renal failure was the most common complication of wet beriberi, with some of the patients requiring CRRT.^[2,5,8–10,14,16,18–22]^ Watson et al^[30]^ reported that 39.4% (13/33) of wet beriberi patients had acute renal failure with high levels of blood lactate and pyruvate, which produced peripheral arteriovenous shunts, renal vascular contraction and blood flow reduction, resulting in a decreased glomerular filtration rate. Acute liver failure is another complication associated with wet beriberi.^[14,16,19,20]^ Watson et al^[30]^ reported that 18.8% (6/32) of wet beriberi patients had acute liver failure related to hepatic congestion caused by right heart failure. In addition to acute renal failure and liver failure, our patient also had coagulation disorders and electrolyte disturbances (severe hyponatremia). Furthermore, measurements of serum thiamine concentration, the TPP effect and erythrocyte transketolase activity were the most common methods used, especially the measurement of thiamine concentration, in documents discussing the diagnosis of wet beriberi (Table 4). Notably, there was lack of sensitivity and specificity data on these tests.

**Table 3:**
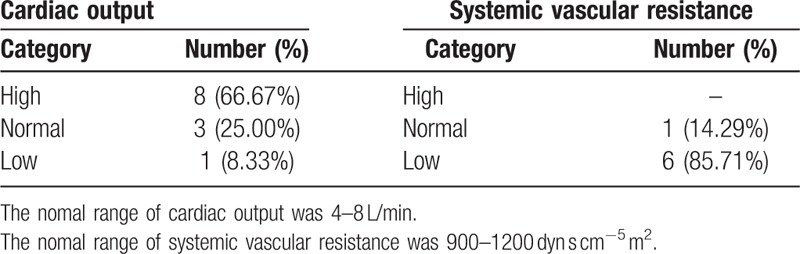
Reports of cardiac output and systemic vascular resistance.

**Table 4:**
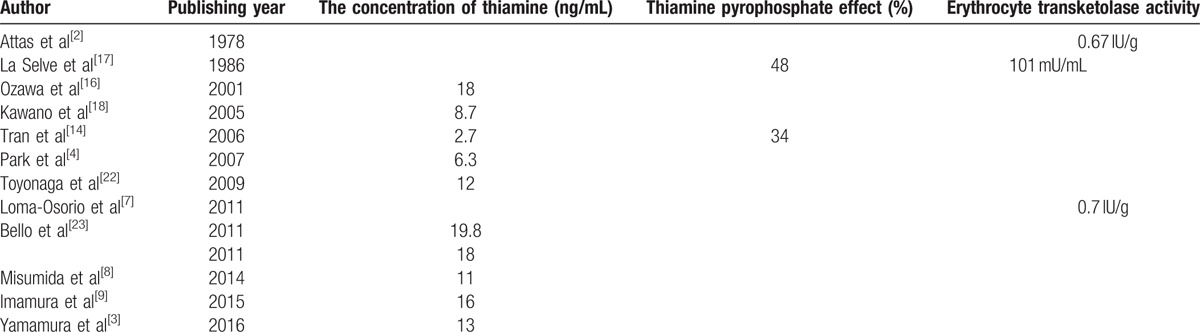
Currently available diagnostic tests for wet beriberi.

Treatment with vitamin B1 is useful for the diagnosis and treatment of wet beriberi. It is generally accepted that suspected patients should be given a therapeutic administration of thiamine. Because measurement of serum thiamine concentration is difficult, complex, and uncommon, the results often arrive late and lack specificity; however, treatment with thiamine is considered safe. Signs of poisoning have not been reported when the blood concentration of thiamine was very high. Wrenn et al^[31]^ retrospectively studied 989 patients receiving intravenous administration of vitamin B1 in a 100-mg bolus and found only 1 case of pruritus (major reaction) and 11 cases of transient local irritation (minor reaction).

Administration of thiamine for wet beriberi varies among authors (Table 5). Generally, alcohol-related beriberi patients were administered a higher dose of thiamine than non-alcohol-related beriberi patients, because alcohol can inhibit the uptake of vitamin B1 and the phosphorylation of its active form (TPP).^[12,32]^ For non-alcohol-related wet beriberi patients, currently, the most common treatment is a daily intravenous treatment of 100 to 200 mg of thiamine. Additionally, the dose of thiamine should be increased based on the presence of life-threatening conditions;^[12]^ later, oral administration is indicated. For alcohol-related beriberi patients, doses should be increased appropriately.

**Table 5:**
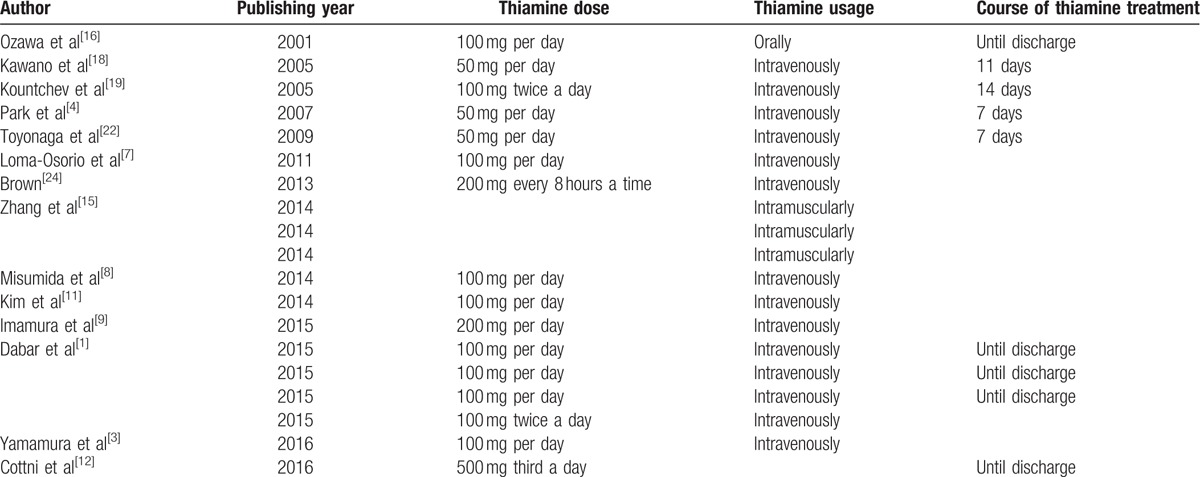
Reports of thiamine use.

For Shoshin beriberi patients, after thiamine treatment, the hemodynamic indices (blood pressure, pulse, urine volume, serum lactate, etc.) dramatically improved in minutes to hours,^[1,3,7–9,11,12,16,18]^ and we were able to rapidly reduce and finally stop vasopressor treatment in the following hours, as the hemodynamic indices returned to normal values.^[1,9,11,19]^ For our patient, the hemodynamic indices improved within 12 hours, and we stopped vasopressor treatment in the following 48 hours.

## 4 Conclusion

We have presented a case of wet beriberi (Shoshin beriberi) associated with MOF in a prison patient with a history of heavy alcohol consumption, an especially causal factor in this disease, in order to draw attention to etiology, typical features, diagnostic tests, and thiamine use for this forgotten but memorable disease. Patients with malnourished diet who have unexplained heart failure, lactic acidosis, and/or MOF should be empirically given thiamine administration without delay.

## Supplementary Material

Supplemental Digital Content

## References

[1] Dabar G Harmouche C Habr B Shoshin Beriberi in critically-Ill patients: case series. Nutr J 2015;14:51. 25982313 10.1186/s12937-015-0039-7 PMC4443551

[2] Attas M Hanley HG Stultz D Fulminant beriberi heart disease with lactic acidosis: presentation of a case with evaluation of left ventricular function and review of pathophysiologic mechanisms. Circulation 1978;58:566–72. 679449 10.1161/01.cir.58.3.566

[3] Yamamura M Murai H Kaneko S Case report: pericardial effusion with constrictive physiology in a patient with wet beriberi. Nutr J 2016;15:37. 27059308 10.1186/s12937-016-0156-y PMC4826515

[4] Park JH Lee JH Jeong JO Thiamine deficiency as a rare cause of reversible severe pulmonary hypertension. Int J Cardiol 2007;121:e1–3. 17346820 10.1016/j.ijcard.2006.08.054

[5] Pereira VG Masuda Z Katz A Shoshin beriberi: report of two successfully treated patients with hemodynamic documentation. Am J Cardiol 1984;53:1467. 6720597 10.1016/s0002-9149(84)91365-1

[6] Akpan T Peschard S Brinkane AH [Right heart failure caused by thiamine deficiency (cardiac beriberi)]. Presse Med 2000;29:240–1. 10701400

[7] Loma-Osorio P Penafiel P Doltra A Shoshin beriberi mimicking a high-risk non-ST-segment elevation acute coronary syndrome with cardiogenic shock: when the arteries are not guilty. J Emerg Med 2011;41:e73–7. 18930369 10.1016/j.jemermed.2008.03.040

[8] Misumida N Umeda H Iwase M Shoshin beriberi induced by long-term administration of diuretics: a case report. Case Rep Cardiol 2014;2014:878915. 25105030 10.1155/2014/878915 PMC4106092

[9] Imamura T Kinugawa K Shoshin Beriberi with low cardiac output and hemodynamic deterioration treated dramatically by thiamine administration. Int Heart J 2015;56:568–70. 26346515 10.1536/ihj.15-033

[10] Chisolm-Straker M Cherkas D Altered and unstable: wet beriberi, a clinical review. J Emerg Med 2013;45:341–4. 23849362 10.1016/j.jemermed.2013.04.022

[11] Kim J Park S Kim JH A case of shoshin beriberi presenting as cardiogenic shock with diffuse ST-segment elevation, which dramatically improved after a single dose of thiamine. Cardiovasc J Afr 2014;25:e1–5. 10.5830/CVJA-2014-053 25625639

[12] Cottini M Ranucci M Facciolo C An unusual case of cardiogenic shock in which thiamine administration led to reversal of lactic acidosis and heart function recovery: Shoshin beriberi in an adolescent. Int J Cardiol 2016;222:401–3. 27505323 10.1016/j.ijcard.2016.07.248

[13] Ward KE Happel KI An eating disorder leading to wet beriberi heart failure in a 30-year-old woman. Am J Emerg Med 2013;31:460.e5–6. 10.1016/j.ajem.2012.08.007 23158607

[14] Tran HA A 74-year-old woman with increasing dyspnea. Wet berberi with fulminant (Shoshin) cardiac failure and elevated troponin I. Arch Pathol Lab Med 2006;130:e8–10. 16390253 10.5858/2006-130-e8-AYWWID

[15] Zhang JJ Chen YH Ju YF Three cases of beriberi heart disease analysis. Heilongjiang Med J 2014;38:812–3. (in Chinese).

[16] Ozawa H Homma Y Arisawa H Severe metabolic acidosis and heart failure due to thiamine deficiency. Nutrition 2001;17:351–2. 11369178 10.1016/s0899-9007(00)00588-8

[17] La Selve P Demolin P Holzapfel L Shoshin beriberi: an unusual complication of prolonged parenteral nutrition. JPEN J Parenter Enteral Nutr 1986;10:102–3. 3080618 10.1177/0148607186010001102

[18] Kawano H Koide Y Toda G ST-segment elevation of electrocardiogram in a patient with Shoshin beriberi. Intern Med 2005;44:578–85. 16020883 10.2169/internalmedicine.44.578

[19] Kountchev J Bijuklic K Bellmann R A patient with severe lactic acidosis and rapidly evolving multiple organ failure: a case of shoshin beri-beri. Intensive Care Med 2005;31:1004. 15875157 10.1007/s00134-005-2648-7

[20] Yamasaki H Tada H Kawano S Reversible pulmonary hypertension, lactic acidosis, and rapidly evolving multiple organ failure as manifestations of Shoshin Beriberi. Circ J 2010;74:1983–5. 20622473 10.1253/circj.cj-10-0202

[21] Smith SW Severe acidosis and hyperdynamic circulation in a 39-year-old alcoholic. J Emerg Med 1998;16:587–91. 9696175 10.1016/s0736-4679(98)00040-7

[22] Toyonaga J Masutani K Tsuruya K Severe anasarca due to beriberi heart disease and diabetic nephropathy. Clin Exp Nephrol 2009;13:518–21. 19459028 10.1007/s10157-009-0189-z

[23] Bello S Neri M Riezzo I Cardiac beriberi: morphological findings in two fatal cases. Diagn Pathol 2011;6:8. 21244717 10.1186/1746-1596-6-8 PMC3034660

[24] Brown TM A case of Shoshin Beriberi: lessons old and new for the psychiatrist. Psychosomatics 2013;54:175–80. 22658327 10.1016/j.psym.2012.01.010

[25] Lee HS Lee SA Shin HS A case of cardiac beriberi: a forgotten but memorable disease. Korean Circ J 2013;43:569–72. 24044018 10.4070/kcj.2013.43.8.569 PMC3772304

[26] Punchai S Hanipah ZN Meister KM Neurologic manifestations of vitamin B deficiency after bariatric surgery. Obes Surg 2017;27:2079–82. 28213665 10.1007/s11695-017-2607-8

[27] Cisse FA Konate MM Ekue WA Clinical appearance and scalable profile Thiamine deficiency in prison in Guinea: study of thirty-eight observations. Bull Soc Pathol Exot 2016;109:70–6. 27100861 10.1007/s13149-016-0484-3

[28] Roman-Campos D Cruz JS Current aspects of thiamine deficiency on heart function. Life Sci 2014;98:1–5. 24398040 10.1016/j.lfs.2013.12.029

[29] Hanninen SA Darling PB Sole MJ The prevalence of thiamin deficiency in hospitalized patients with congestive heart failure. J Am Coll Cardiol 2006;47:354–61. 16412860 10.1016/j.jacc.2005.08.060

[30] Watson JT El Bushra H Lebo EJ Outbreak of beriberi among African Union troops in Mogadishu, Somalia. PLoS One 2011;6:e28345. 22205947 10.1371/journal.pone.0028345 PMC3244391

[31] Wrenn KD Murphy F Slovis CM A toxicity study of parenteral thiamine hydrochloride. Ann Emerg Med 1989;18:867–70. 2757284 10.1016/s0196-0644(89)80215-x

[32] Galvin R Brathen G Ivashynka A EFNS guidelines for diagnosis, therapy and prevention of Wernicke encephalopathy. Eur J Neurol 2010;17:1408–18. 20642790 10.1111/j.1468-1331.2010.03153.x

