# Supplementary material for: Wet beriberi with multiple organ failure remarkably reversed by thiamine administration: A case report and literature review
Source: Medicine (Baltimore). 2018 Mar 2;97(9):e0010. doi: 10.1097/MD.0000000000010010 (PMC5851725; doi:10.1097/MD.0000000000010010)
Supplement: Supplemental Digital Content [file medi-97-e0010-s001.doc]

| **Supplementary table 1**. The sequential date of serum lactate. | | | | | |
| --- | --- | --- | --- | --- | --- |
| Time | On admission | Day 2 | Day 3 (started intramuscular thiamine) | Day 4 | Day 8 |
| Lactate  (mmol/L) | 5 | 5.6 |  | 1.7 | 1.1 |

The reference range of lactate was 0.7-2.1 mmol/L.
